# ID 133 - Perfil de Eventos Adversos dos Medicamentos Utilizados no Tratamento das Leishmanioses no Brasil (2018-2023)

**DOI:** 10.5327/2237-9622.2025.v34s1.133

**Published:** 2025-11-25

**Authors:** Mônica Cristiane Rodrigues, Adriane Lopes Medeiros Simone, Bruna Bento dos Santos, Stéfani Sousa Borges, Daniela Oliveira de Melo

**Keywords:** leishmanioses, eventos adversos, farmacovigilância

## Abstract

**Introdução:**

A leishmaniose é uma doença zoonótica que pode causar diversas síndromes clínicas em humanos, comprometendo a pele, as membranas mucosas e as vísceras. O tratamento deve ser específico para cada forma clínica, levando em consideração as características individuais do paciente e o perfil de segurança dos medicamentos, a fim de reduzir a mortalidade associada à doença. O objetivo foi avaliar o perfil de eventos adversos (EA) dos medicamentos disponíveis e utilizados no Brasil.

**Método:**

Foram coletados dados abertos de notificação da plataforma de farmacovigilância VigiMed da Agência Nacional de Vigilância Sanitária (Anvisa), do período de 2018 a 2023, sobre a suspeita de EA a medicamentos recomendados no do Ministério da Saúde (MS) do Brasil (2024). As variáveis consideradas foram sexo, faixa etária, unidade federativa de notificação, tipo de notificador e os cinco principais EA associados a cada medicamento. Foi feita uma filtragem para identificar padrões de notificação.

**Resultados:**

O protocolo de tratamento para leishmaniose visceral (LV) recomenda o antimoniato de meglumina (AM) e a anfotericina B lipossomal (L-AMB). Para leishmaniose tegumentar (LT), além desses dois, inclui o isetionato de pentamidina (IP), para a forma cutânea, e a miltefosina, para casos de coinfecção com HIV com falha terapêutica. Dados do VigiMed mostraram um total de 698 notificações de suspeita de EA: 217 para AM, 8 para IP e 473 para anfotericinas (319 para anfotericina B, 24 para complexo lipídico, 129 para L-AMB e 1 para desoxicolato). Não houve notificação para a miltefosina. Os registros foram principalmente da Região Sudeste, com Minas Gerais respondendo por 84,79% (184/217) das notificações de AM e 34% (44/129) de L-AMB. A maioria das notificações envolveu o sexo masculino, com idade entre 18 e 65 anos, e foi realizada por farmacêuticos, com um aumento notável no número de registros a partir de 2020.

**Conclusão:**

Em 2022, foram publicadas as "Diretrizes para o Tratamento da Leishmaniose nas Américas" pela Organização Pan-Americana da Saúde, recomendando o uso prioritário da L-AMB para imunocompetentes e imunocomprometidos devido ao seu melhor perfil de segurança e adesão. No Brasil, entretanto, o AM continua sendo a primeira escolha de tratamento, sendo possível a administração ambulatorial, apesar do volume de notificações de suspeitas de EAs. A L-AMB é usada exclusivamente em ambiente hospitalar devido à necessidade de monitoramento rigoroso da toxicidade, porém ainda está restrita a determinadas populações. O perfil de distribuição de casos por sexo e faixa etária é semelhante ao relatado pela área técnica do MS, acometendo a população adulta economicamente ativa, majoritariamente do sexo masculino e crianças menores que 10 anos. Ao contrário do esperado, a distribuição geográfica dos casos não revela concentração nas Regiões Norte e Nordeste, o que sugere subnotificação e/ou preenchimento incorreto das notificações. Embora a análise de dados secundários tenha limitações, conclui-se que há necessidade de explorar novas opções terapêuticas para LV e LT, considerando a diversidade geográfica e as necessidades da população, além de reforçar a sensibilização dos profissionais de saúde para melhorar a vigilância e manejo das leishmanioses.

